# The Evolution of Dilatant Shear Bands in High-Pressure Die Casting for Al-Si Alloys

**DOI:** 10.3390/ma17205001

**Published:** 2024-10-12

**Authors:** Jingzhou Lu, Ewan Lordan, Yijie Zhang, Zhongyun Fan, Wanlin Wang, Kun Dou

**Affiliations:** 1 School of Metallurgy and Environment, Central South University, Changsha 410083, China; 213506005@csu.edu.cn (J.L.);; 2 Brunel Centre for Advanced Solidification Technology (BCAST), Brunel University London, Kingston Lane, Uxbridge UB8 3PH, UK; ewan.lordan@brunel.ac.uk (E.L.); robertzyj@163.com (Y.Z.); zhongyun.fan@brunel.ac.uk (Z.F.)

**Keywords:** shear bands, segregation, defects, aluminium alloys, high-pressure die casting

## Abstract

Bands of interdendritic porosity and positive macrosegregation are commonly observed in pressure die castings, with previous studies demonstrating their close relation to dilatant shear bands in granular materials. Despite recent technological developments, the micromechanism governing dilatancy in the high-pressure die casting (HPDC) process for alloys between liquid and solid temperature regions is still not fully understood. To investigate the influence of fluid flow and the size of externally solidified crystals (ESCs) on the evolution of dilatant shear bands in HPDC, various filling velocities were trialled to produce HPDC samples of Al8SiMnMg alloys. This study demonstrates that crystal fragmentation is accompanied by a decrease in dilatational concentration, producing an indistinct shear band. Once crystal fragmentation stagnates, the enhanced deformation rate associated with a further increase in filling velocity (from 2.2 ms^−1^ to 4.6 ms^−1^) localizes dilatancy into a highly concentrated shear band. The optimal piston velocity is 3.6 ms^−1^, under which the average ESC size reaches the minimum, and the average yield stress and overall product of strength and elongation reach the maximum values of 144.6 MPa and 3.664 GPa%, respectively. By adopting the concept of force chain buckling in granular media, the evolution of dilatant shear bands in equiaxed solidifying alloys can be adequately explained based on further verification with DEM-type modeling in OpenFOAM. Three mechanisms for ESC-enhanced dilation are presented, elucidating previous reports relating the presence of ESCs to the subsequent shear band characteristics. By applying the physics of granular materials to equiaxed solidifying alloys, unique opportunities are presented for process optimization and microstructural modeling in HPDC.

## 1. Introduction

High-pressure die casting is a popular manufacturing process for light metals and is known for its high productivity, high dimensional accuracy, and excellent mechanical properties. However, fierce turbulence occurs during the injection phase due to the rapid filling speed and narrow ingate of the casting system. The *J* factor [Equation (1)], which indicates the initial flow regime during filling, has received considerable attention in Japan, with recent studies aiming to establish the relation between its value and the subsequent product’s quality [1,2,3]. The *J* factor can be calculated using the VanRens equation:

(1) $$J=D∙\rho ∙V_{g}^{1.71}$$

where *V_g_* is the gate velocity, *D = ab*/(*a + b*) is the orifice radius, *a* is the gate width, *b* is the gate thickness, and *ρ* is the density of the alloy [3]. By capturing the high-speed ejection of molten metal into an open space whilst irradiating laser light, researchers have proven that the flow regime becomes partial atomization during mould filling when the calculated *J* factor reaches a high value [4]. Atomization involves two distinct aspects: (i) The collision between the newly accelerated high-speed material and the previously injected low-speed material results in the front of the liquid jet expanding in an umbrella-like manner. This phenomenon gives rise to the generation of molten metal droplets, with a size range equivalent to the Sauter mean diameter, emanating from its perimeter. (ii) The surface fluctuations within the liquid jet result in the detachment of droplets of molten metal from the liquid column [4,5], which occurs instantly before the melt temperature drops noticeably, and can be treated as an isothermal process. The numerical modeling of the atomization phenomenon, achieved by a coupled large eddy simulation and the volume of the fluid approach, suggests that this atomized material is conveyed into the cavity as a multiphase flow containing finely dispersed air bubbles, persisting in this manner until filling is complete [5].

Intensive melt shearing, such as that introduced in the runner and ingate at the time of injection, has been shown to significantly influence the morphology and distribution of coarse externally solidified crystals (ESCs) observed in the final microstructure [6,7,8,9,10]. Wu et al. reported that an increase in the filling velocity led to the fragmentation and re-melting of crystals for an AM60B magnesium alloy, attributed to induced thermal shock and convection within the melt [9]. Li et al. discussed the rotation and fragmentation of crystals in relation to the aggregation of porosity within defect bands for an AZ91D magnesium alloy [10]. Gourlay et al. demonstrated that ESCs are not a prerequisite for the formation of defect bands, although their presence likely influences the rheology of the solidifying alloy, altering the characteristics of the residual band [11].

Previous investigations into vane rheometry and direct shear cells have indicated that the rheological behavior of equiaxed solidifying alloys can be conceptualized as resembling cohesionless, compacted particle materials [12,13]. The grains within compacted particle materials react to shear/compact forces by re-organizing to generate bands of dilation and localized contraction. In the equiaxed zone for light alloys such as Al–Si–Mg and Mg–Al–Z during the solidification process, the increase in shear stress is always followed by the expansion in bulk volume, which is termed Reynold’s dilatancy [14]. During the stage of increasing shear stress, the dilatancy changes rapidly in an anisotropic way, before finally forming a localized narrow band, which follows the filling direction within the casting. The typical thickness of the shear bands found in HPDC parts has been estimated to fall within a scale of 7 to 18 mean grains in width, which aligns with the characteristic width range of 6 to 20 mean grains for dilatant shear bands occurring in particle materials [15]. It is anticipated that the low crystal packing density of the band will lead to the occurrence of positive macrosegregation, provided there is an adequate supply of liquid to be drawn towards the expanding band. Conversely, in instances where there is insufficient liquid available, interdendritic porosity is likely to occur [14]. Previous studies have suggested that the HPDC process parameters influence the formation and characteristics of shear bands, with a key focus on controlling the intensification stage and the thermal conditions in the shot sleeve; however, few have pondered the influence of flow conditions on the band characteristics [8,12,16,17].

Tordesillas characterizes the relation of stress–dilatancy in particle materials via discrete element simulation (DEM). Their results have shown that the potential mechanism is a cycle-like behavior of jamming–unjamming, which is controlled by the accumulating buckling of “force chains” [18,19]. In our work, we introduce the implications of force chain buckling on dilatant shear banding in equiaxed solidifying alloys, presenting three novel mechanisms for ESC-enhanced dilation in HPDC. The influences of several filling velocities on the evolution of dilatant shear bands in HPDC will be discussed in relation to the initial jet flow conditions and the subsequent crystal morphology.

## 2. Materials and Methods

### 2.1. Materials

A total of 35 Kg of Al8SiMnMg alloy (liquidus temperature of 615 °C) was melted in an electric resistance furnace and then held at 750 °C for 30 min to maintain homogenization. The molten alloy was degassed using the rotating equipment for 10 min, and the rotation speed was 350 rounds per minute with an Ar gas supply rate of 4 L per minute.

### 2.2. Casting Technologies and Parameters

**Figure 1:**
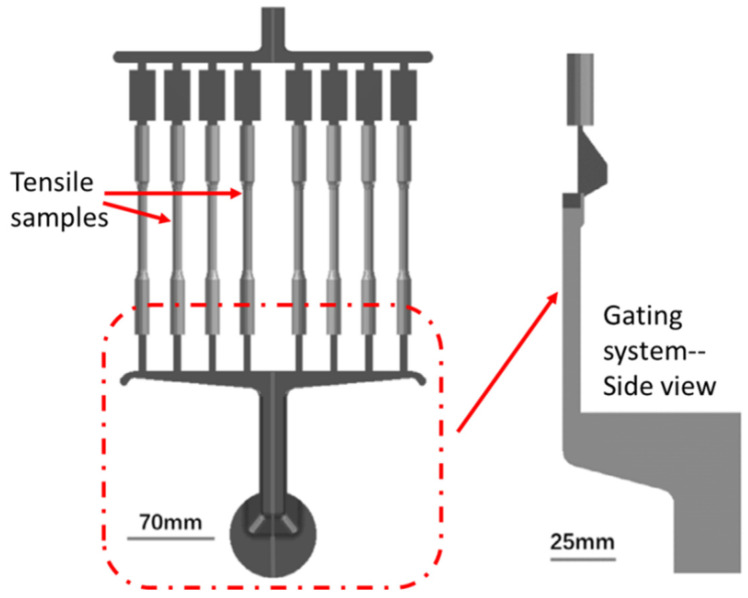
Casting region with gating system and 8 tensile samples of HPDC tensile.

**Figure 2:**
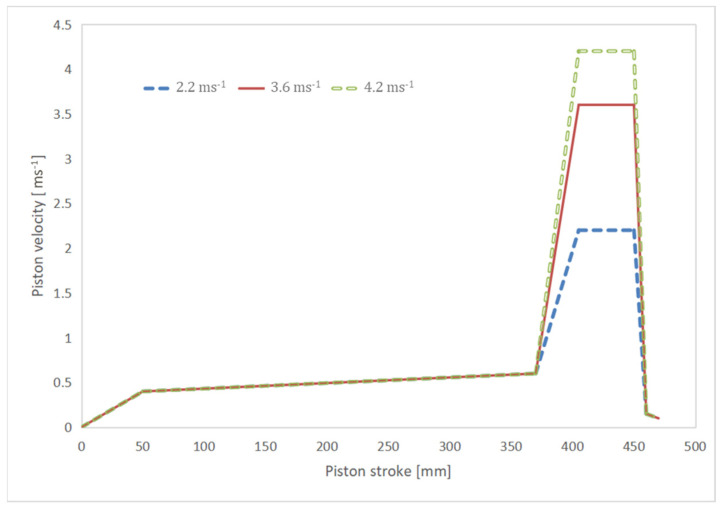
Shot profile highlighting filling velocities used to produce HPDC tensile specimens.

During HPDC, the melted alloy was manually dosed into the shot chamber of 450 t cold chamber HPDC equipment using a transferring crucible. The temperatures of the molten alloy, shot chamber, and casting chamber were, respectively, maintained at 680 °C, 180 °C, and 150 °C, ensuring a precise and controlled environment for the entire process. The melt was subsequently infused into the casting chamber, employing filling velocities of 2.2 ms^−1^, 3.6 ms^−1^, and 4.2 ms^−1^, respectively. This process yielded eight circular tensile specimens, each possessing a nominal gauge diameter of ø6.35 mm, fully adhering to the stipulated ASTM standards [20]. Once the cavity was completely filled, an intensification pressure of 60 MPa was applied. A detailed description of the die geometry used to produce these tensile specimens can be found in [21] and is shown in Figure 1, and the plunger velocity curves applied in this paper are shown in Figure 2. The entire piston stroke was 470 mm, and, during the initial shot stages for the three velocity profiles, the piston velocity increased from 0 to 0.4 ms^−1^ and then gradually to 0.6 ms^−1^. During the fast shot stage, the three velocity profiles differed in terms of the filling speed, as mentioned above. The reasons for the selection of characteristic filling velocities of 2.2 ms^−1^, 3.6 ms^−1^, and 4.2 ms^−1^ are described in detail in previous works of the authors [22,23].

**Figure 3:**
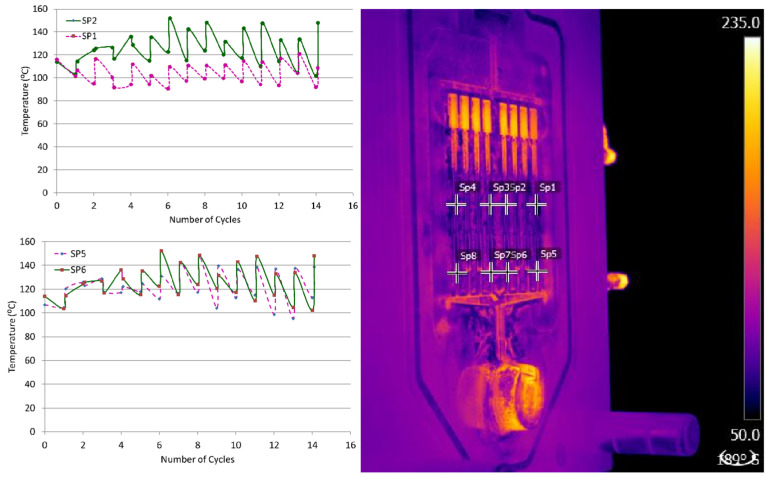
Infrared image and the temperature readings during various cycles of the HPDC process.

To maintain consistent die temperature, a FLIR T650sc infrared camera was utilized to acquire the thermal-image data. From the infrared images, temperature readings were taken from the center of the gage section and at the ingate using point measurements within the thermographic post-processing software FLIR tools (version FLIR Tools+ 6.4.18039.1003). An illustration of the infrared images and the temperature readings are shown in Figure 3. It was found that after six shots, a steady-state die temperature was achieved. The first six shots were therefore scrapped, and samples were taken from the proceeding shots.

### 2.3. Tensile Test

Tensile tests were conducted at room temperature utilizing the Instron 5500 universal electromechanical testing system, adhering strictly to the ASTM standard E8/E8M [20]. The gauge length and gauge diameter of the tensile specimens were precisely measured to be 55 mm and 6.35 mm, respectively. The tensile data were systematically tracked by employing a 50 mm extensometer, with a ramp rate of 1 mm/min, ensuring accuracy and reliability in the testing process.

### 2.4. Microstructure Characterization

Samples for microstructural characterization were extracted from the central portion of the tensile specimens, positioned perpendicularly to the tensile direction. The specimens underwent grinding and polishing procedures to achieve the surface finish of 1 µm, adhering to standard metallographic guidelines. Subsequently, to uncover the microstructural features, the samples were etched using Keller’s reagent. Optical microscopy and scanning electron microscopy (SEM) were used to observe the etched microstructures of the HPDC samples. High-contrast SEM micrographs for grain size measurements were obtained via a tungsten filament Carl Zeiss LEO 1455VP SEM (ZEISS, Jena, Germany) and analyzed using image processing packages such as ImageJ https://imagej.net/ij/ (accessed on 8 October 2024).

## 3. Results

### 3.1. Dilatant Shear Bands and ESC Morphology

**Figure 4:**
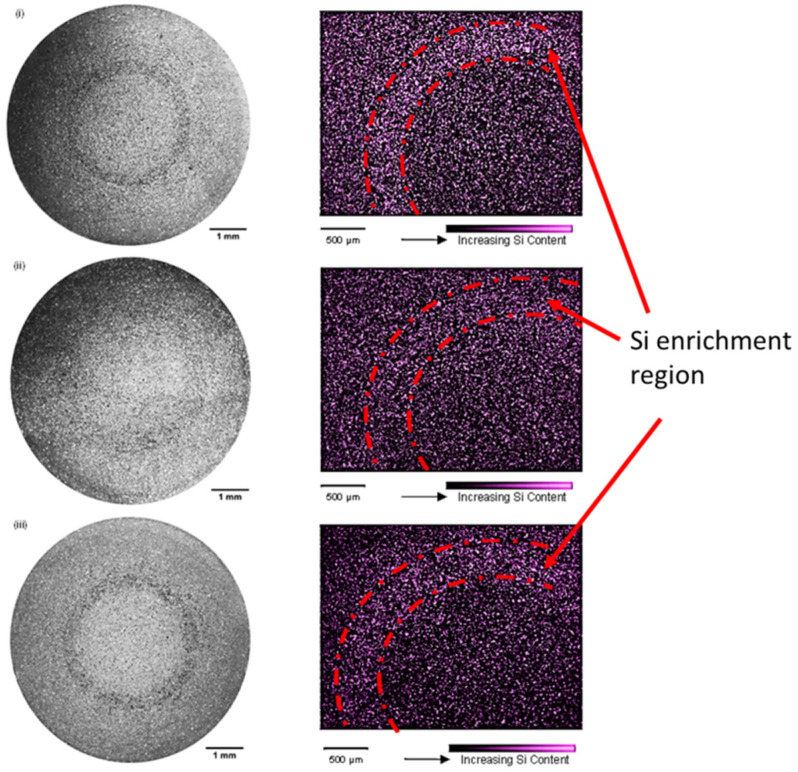
Dilatant shear bands observed in HPDC samples produced with filling velocities of 2.2 ms^−1^ (**i**), 3.6 ms^−1^ (**ii**) and 4.2 ms^−1^ (**iii**). Typical macrostructure of etched samples from the center of the gage section are shown (left) and corresponding EDX maps highlighting the eutectic fraction are shown (right).

Bands of positive macrosegregation were observed with all of the filling velocities trialled in this study (Figure 4). With an increase in velocity from 2.2 ms^−1^ to 3.6 ms^−1^, dilatational concentration (defined here as the degree of local dilation, thus characterized by the local eutectic fraction) was observed to decrease, suggesting a transition from localized dilation within a shear band to global dilation of the entire assembly. Further increasing the filling velocity from 3.6 ms^−1^ to 4.2 ms^−1^ once again produced a distinct shear band. The normalized width of the Si enrichment region in Figure 3 (red dash line area) was estimated in ImageJ software and the normalized values were 0.92, 0.52 and 0.85, respectively, when filling speed increased from 2.2 ms^−1^ to 4.2 ms^−1^.

**Figure 5:**
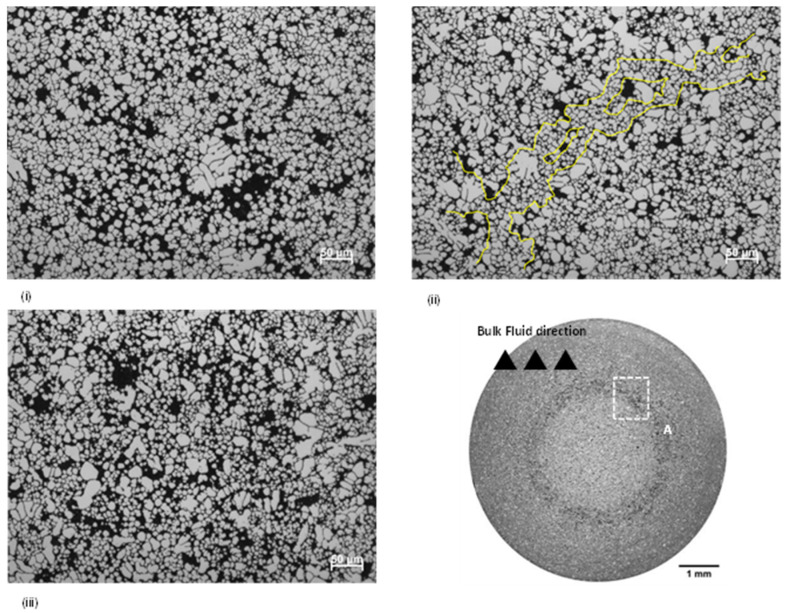
Optical micrographs taken from zone A, showing how dilatancy varies with filling velocities of (**i**) 2.2 ms^−1^, (**ii**) 3.6 ms^−1^, and (**iii**) 4.2 ms^−1^. Outlined in (**ii**) is a potential force chain that has persisted through deformation. The bulk filling direction was out of the page.

For samples produced with filling velocities of 2.2 ms^−1^ and 4.2 ms^−1^, large fluctuations in eutectic fraction were observed within the band, reminiscent of the large voids attributed to force chain buckling in granular media (Figure 5). Outlined in Figure 5ii is a columnar crystal structure residing in the shear band of a sample produced at 3.6 ms^−1^, observed to offset slightly from the major principle stress axis (i.e., radial compression during intensification).

### 3.2. Grain Size and Mechanical Properties

**Figure 6:**
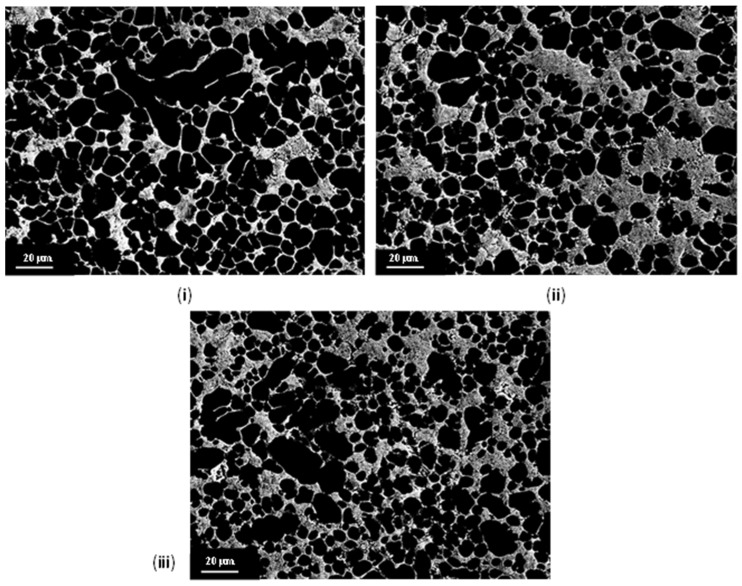
Typical high-contrast secondary electron SEM micrographs used to obtain average ESC and in-cavity solidified grain size (Table 1) for filling velocities of (**i**) 2.2 ms^−1^, (**ii**) 3.6 ms^−1^ and (**iii**) 4.2 ms^−1^.

High-contrast SEM micrographs captured within the shear band were used to determine the mean diameter of the ESCs and the solidified grains in the casting chamber for samples produced with the aforementioned filling velocities (Table 1). Within the image processing packages, SEM results were treated using an appropriate threshold to separate grains from the background (Figure 6). The mean grain sizes (*Φ*) were determined by applying the equivalent circle method (Equation (2)). Grains with a diameter ≥ 20 µm were defined as ESCs and were separated from in-cavity solidified grains (3 µm ≤ $\phi_{i}$ ≤ 20 µm) for subsequent analysis.

(2) $$Area\;of\;crystal=\pi \phi_{i}/4,\;\phi =\sum {(\phi }_{i}/N_{p})$$

An increase in filling velocity from 2.2 ms^−1^ to 3.6 ms^−1^ resulted in a decrease in the average ESC size from 41 µm to 27 µm. Further increases in velocity from 3.6 ms^−1^ to 4.2 ms^−1^ did not significantly alter the average ESC size, with only a slight increase from 27 µm to 33 µm; however, the considerably reduced average sizes of in-cavity solidified grains were observed in this process, with the mean values having decreased from 8.1 µm to 7.3 µm.

**Figure 7:**
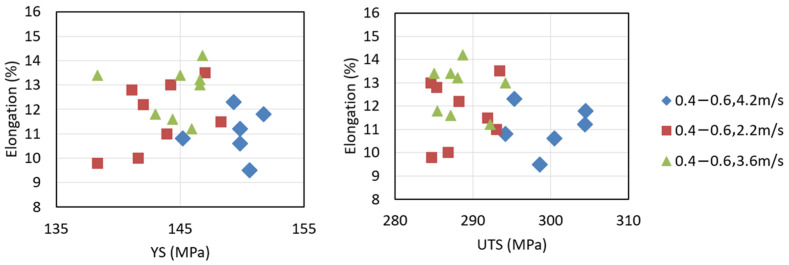
The yield stress, elongation and ultimate tensile stress of various tensile samples.

The yield stress, elongation and ultimate tensile stress of various tensile samples were tested and are plotted in Figure 7. As is shown in the figure, the mechanical properties shown some variability in different locations and under various piston speeds. The average values for the mechanical properties are summarized in Table 2. Combining Table 1 and Table 2, it could be seen that with the piston velocity of 3.6 ms^−1^, the average ESC size reached the minimum value and the average yield stress and overall product of strength and elongation reached the maximum value, of 144.6 MPa and 3.664 GPa%, respectively.

## 4. Discussion

The term “strong network” refers to a subnetwork composed of interconnected chains of particles that are subjected to significant stress levels, thereby assuming the bulk of the applied load. Conversely, the complementary “weak network” comprises contacts that undergo a force level below the average, and serves to provide lateral reinforcement to the existing force chains [18,22]. Whilst Radjai very appropriately states “that the weak network behaves essentially as an interstitial liquid, whereas the strong forces carry the whole deviatoric load and in this respect behaves as a solid”, we cannot simply reverse this analogy to describe microstructural evolution during equiaxed solidification without first considering that the weak network is more likely to comprise a diluted suspension of liquid and dispersed crystals.

The initial deformation of the particle materials is primarily affine in nature, characterized by a corresponding increase in potential energy as strain accumulates [18,19]. From the start of the deformation process, force chains form and accumulate, which are in parallel with the direction of the first principal stress. The disappearance of force chain buckling is followed by bulk dilation of the particle materials [18,19,23]. Force chain buckling begins before the maximum shear stress is reached, causing obvious holes between columns [24,25,26]. After the peak shear stress, force chain buckling propagates, causing dilatancy to localize within a narrow shear band [19]. In the occurrence of a force chain failure, also known as an unjamming event, the energy that had been accumulated at its contact points is released and transmitted to adjacent particles within the weaker network. This process prompts the emergence of novel force chains or the enhancement of those already present, which is referred to as a jamming event [19]. The shear band progresses continuously, until the repetitive process of unjamming and jamming culminates in the band attaining a residual state, wherein the collapse of existing force chains is balanced by the emergence of novel microstructures [25]; however, when deliberating dilatancy in equiaxed solidifying alloys, one must consider that with sufficiently high cooling rates, the casting may solidify before the band achieves this residual state.

**Figure 8:**
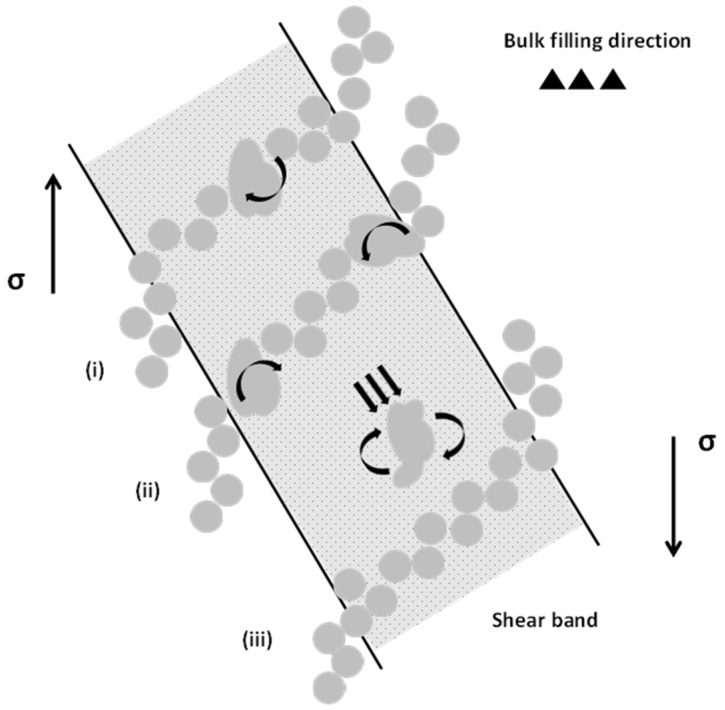
Graphical illustration highlighting the three mechanisms governing ESC enhanced dilation within the shear band: (i) “Stacking faults” introduced by the presence of ESCs along the force chain; (ii) ESCs located on the outermost regions of the band effectively acting as pivots; (iii) ESCs propelled by highly turbulent flow conditions, potentially dislodging crystals from the force chain. σ denotes the major principle stress axis.

Particle rotation during filling has been widely reported, predominantly originating from highly turbulent flow conditions and subsequent particle interactions within the multiphase suspension [10,27,28,29,30]. The size (10~200 µm) and morphology (globular/irregular) of these particles greatly influences the flow of the suspension. In a given velocity gradient, increasing the particle size leads to enhanced rotation and an increased collision frequency [27]. It is evident from (Figure 4 and Figure 5, Table 1) that crystal fragmentation is accompanied by a decrease in dilatational concentration, with the normalized width of the Si enrichment region decreasing from 0.92 to 0.52. Surmising the concept of force chain buckling in equiaxed solidifying alloys, we can explain the influence of fluid flow and crystal morphology on dilatant shear banding in HPDC. Figure 8 illustrates the three novel mechanisms of ESC-enhanced dilation proposed by the authors, as follows:

(1) In Figure 8i, the presence of large crystals within the force chain leads to a decrease in the total number of contacts and increases the volume of interstitial fluid. The force chain is therefore more susceptible to buckling, enhancing dilation within the shear band;
(2) Particles within the shear band experience a transverse lift force, due to the existence of a non-uniform pressure distribution. This lift force encourages crystals to migrate towards the region of lowest shear stress [27] (i.e., the centreline of the band). An increase in particle size is followed with a rise in lift force. According to the lift force models in [31,32], an increase in particle size from 50 to 100 µm will lead to an increase of 2.5 × 10^−9^ N in lift force. Within the band, ESCs that comprise the outermost regions of the force chain will effectively act as pivots (Figure 8ii), with the applied moment resulting from the aforementioned slip-shear lift forces;
(3) Within a given velocity gradient, increasing the particle size will increase the energy dissipated upon collision. In Figure 8iii, a large crystal traveling at high velocity may possess the potential to dislodge a segment of the force chain, provided the energy dissipated upon collision is sufficient to encourage local particle rearrangement. This phenomenon is likely to occur towards the centreline of the shear band where resistance to flow is at a minimum, and particle momentum is likely to be at a maximum.

With a further increase in filling velocity from 3.6 ms^−1^ to 4.2 ms^−1^, the average size of ESCs was observed to remain relatively stable. This suggests that crystal fragmentation stagnates at a threshold J-factor, governed by the ingate geometry. For the ingate geometry used throughout this series of experiments, the filling velocity threshold corresponding to the atomization phenomenon is 0.71 ms^−1^. Therefore, it can be assumed that for all filling velocities trialled in this study, melt flowing into the die cavity consists of a liquid column, encompassing a large proportion of ESCs, and fine droplets that rapidly solidify due to their small volume.

Despite the apparent stability in the average ESC size, dilatancy was observed to once again localize into a distinct shear band for samples produced at 4.2 ms^−1^. This is likely to result from the increased shear stress introduced at 4.2 ms^−1^, increasing the rate of force chain buckling within the band. A considerable refinement of the in-cavity solidified grains from 8.1 µm to 7.3 µm was also observed following the rise in filling velocity from 3.6 ms^−1^ to 4.2 ms^−1^. This was attributed to the atomization phenomenon, wherein a rise in injection velocity is followed by a reduction in droplet size [33].

**Figure 9:**
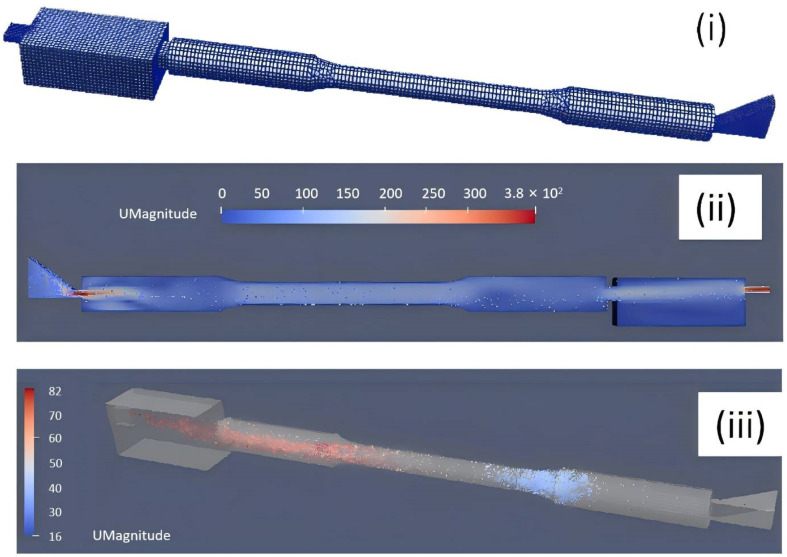
(**i**) Calculation domain and mesh for the model. (**ii**) The fluid velocity distribution of melt. (**iii**) The ESC motion and velocity distribution in the tensile sample during filling.

**Figure 10:**
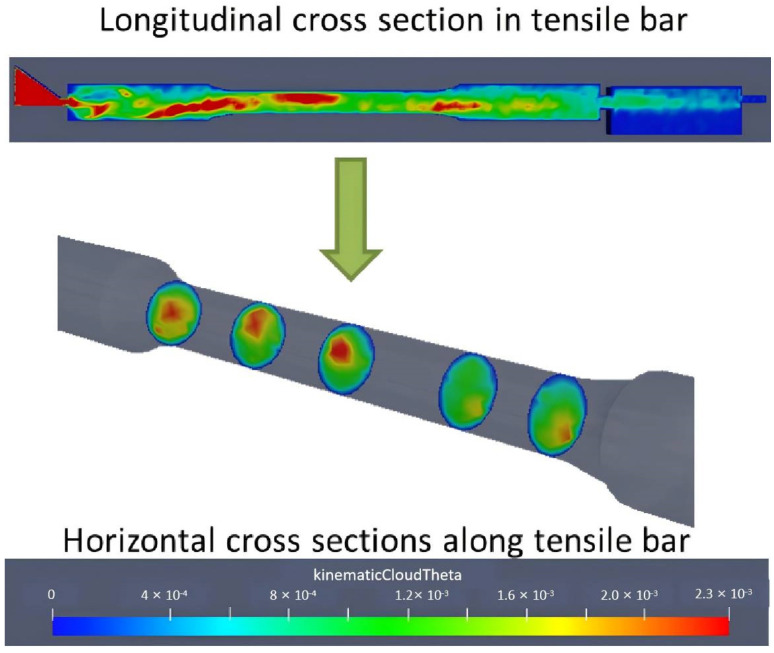
The ESCs’ aggregation tendency in the tensile sample during filling.

To further validate the above mechanism, the melt flow and ESC motion during the HPDC process were modeled based on the OpenFOAM v5.0 platform. Considering similarity, we sought to reduce the computation time of the entire fill system as was shown in Figure 1. A single tensile sample including its ingate and overflow was selected as the modeling region to obtained the characteristic fluid flow and ESC motion behavior. The melt flow behavior is modeled by solving the Navior–Stokes equations and Standard k-epsilon equations. The ESC motion was described using DPMFoam in OpenFOAM. The mesh was generated using blockMesh and snappyHexMesh. Considering that the minimum length for the calculation domain was 2 mm (ingate thickness direction), a meshing characteristic length of 0.5 mm was used for the accurate description of the filling process. The fluid velocity distribution and ESC motion velocity distribution were shown in Figure 9. It was known that during the alloy injection stage, the ESCs were likely to be transported and gather within the central zone along the flow direction in the tensile sample. The ESCs’ aggregation tendency is described in Figure 10. The legend stands for the averaged number density of ESC grains in various positions in a tensile sample. It was clearly observed that this model could accurately predict the evolution of dilatant shear bands in compacted granular assemblies, and this further validated the proposed mechanism for the evolution of dilatant shear bands in the HPDC process for the alloy.

## 5. Conclusions

By adopting the concept of force chain buckling in compacted granular materials, the evolution of dilatant shear bands in equiaxed solidifying alloys can be adequately explained. Fluid flow and grain size are found to significantly influence dilatancy, governing dilatational concentration and subsequently the extent of segregation within the band. The maximum dilatancy of 0.92 (normalized width of the Si enrichment region) is observed when the flow filling speed is 2.2 ms^−1^ and the average ESC size is 41 µm. Increasing the filling velocity appeared to promote crystal fragmentation up until a critical J-factor, leading to a decrease in dilatational concentration and thus a less distinct shear band. Excessive filling velocities increased the deformation rate, leading to an increase in dilatational concentration, and a distinct shear band; however, they also led to the considerable refinement of in-cavity solidified grains, attributed to the atomization phenomenon. To explain the influence of grain size and crystal morphology on the evolution of dilatant shear bands in HPDC, three novel mechanisms for ESC enhanced dilation are presented, as follows:

(1) Stacking faults introduced by the presence of large crystals within the force chain reduce the total number of contacts and increase the volume of interstitial fluid, weakening the structure;
(2) Crystals located towards the outermost region of the band are drawn inwards by slip–shear lift forces. A force chain comprising large crystals at its pivots will experience a greater moment, increasing susceptibility to buckling;
(3) A larger crystal travelling at increasing piston velocity from 2.2 ms^−1^ to 4.2 ms^−1^ may possess the potential to dislodge a segment of a given force chain, provided the energy dissipated upon collision is sufficient to incite local particle rearrangements. The optimal piston velocity is 3.6 ms^−1^, under which the average ESC size reaches the minimum and the average yield stress and overall product of strength and elongation reach the maximum values of 144.6 MPa and 3.664 GPa%, respectively;
(4) Force chain buckling has been studied with sophisticated DEM-type simulations using OpenFOAM platform, which is able to accurately predict the evolution of dilatant shear bands in compacted granular assemblies. By applying the physics of granular materials to equiaxed solidifying alloys, unique opportunities are presented for process optimization and microstructure modeling once the further testing of other alloys has been conducted.

## Figures and Tables

**Table 1:** Average ESC and in-cavity solidified grain sizes within the shear band. All values were calculated using the Fiji (ImageJ) software from high-contrast secondary electron SEM micrographs, such as those shown in Figure 4.

Filling Velocity (ms^−1^)	Average ESC Φ (µm)	Average in Cavity Grain Φ (µm)
2.2	41	8.1
3.6	27	8.1
4.2	33	7.3

**Table 2:** Summary of mechanical properties under various piston velocities.

Average				
Casting Parameters	YS (MPa)	UTS (MPa)	Elongation [%]	Product of Strength and Elongation (GPa%)
0.4–0.6, 2.2 m/s	143.0	288.5	11.5	3.318
0.4–0.6, 3.6 m/s	144.6	288.5	12.7	3.664
0.4–0.6, 4.2 m/s	143.7	292.8	11.3	3.309

## Data Availability

The original contributions presented in the study are included in the article; further inquiries can be directed to the corresponding author.
